# Comments on “Anatomical and visual outcomes of fovea-sparing internal limiting membrane peeling with or without inverted flap technique for myopic foveoschisis”

**DOI:** 10.1186/s12886-023-02963-9

**Published:** 2023-06-14

**Authors:** Yi-Ting Hsieh, Jih-Pin Lin, Chung-May Yang

**Affiliations:** 1 Department of Ophthalmology, National Taiwan University Hospital, 7 Zhongshan S. Rd., Zhongzheng Dist, Taipei, 10002 Taiwan; 2 College of Medicine, National Taiwan University, Taipei, Taiwan; 3 Universal Eye Center, Shangru, Tainan, Taiwan

Dear Editor:

We read with interest the article “Anatomical and visual outcomes of fovea‑sparing internal limiting membrane (ILM) peeling with or without inverted flap technique for myopic foveoschisis” by Zheng et al. [1]. In this article, the authors compared two different surgical techniques for treating myopic foveoschisis, and found that fovea‑sparing ILM peeling, either with or without inverted flap, resulted in similar anatomical and visual outcomes. These results are consistent with our previous study [2]. However, in our study, we found a significantly lower incidence of post-operative macular hole formation in the subgroup of myopic tractional maculopathy (MTM) with lamellar macular hole (LMH) in the combined fovea-sparing ILM peeling and inverted ILM flap group [2]. In Zheng et al’s article, the authors found that in each group, there was one case of MH after surgery as shown in Fig. 3^1^. From the OCT images presented, the case in the inverted ILM flap group had MTM with lamellar macular hole (LMH); after surgery, the OCT images shows a flap closure configuration, instead of a true MH. This pattern is precisely what we would like to achieve with the addition of inverted ILM flap. Bonińska et al. [3] has reported that for flap closure image pattern, the gap usually closed eventually without further intervention. We also have presented the sequential OCT images showing such flap closure pattern in the combined fovea-sparing ILM peeling and inverted ILM flap group in a case of LMH in our previous study [4]. This post-operative OCT configuration highlights the possibility of MH formation after surgery and the advantage of inverted ILM flap in preventing a true MH formation in MTM with LMH. We also believe it may prevent MH formation after surgery in MTM with thin inner roof, even without LMH. Several factors may contribute to the post-operative MH formation after fovea-sparing ILM peeling for MTM: (1) an undetected micro-MH that has existed before the operation; (2) intra-operative manipulation on the thin fovea, such as in eyes with LMH or MTM with a thin foveal lining tissue, as in those with an outer gap and a thin inner roof, even without LMH; (3) asymmetric traction at the slope of posterior staphyloma; and (4) foveal dehiscence due to post-operative residual parafoveal traction. In all these scenarios, an inverted ILM flap may be beneficial to prevent post-operative MH. Given our interpretation of Fig. 3 and the small case number in the study by Zheng et al., [1] we think the conclusion made by them may require some modifications.

## Data Availability

Not applicable.

## References

[1] Zheng D, Huang Z, Zeng Q (2022). Anatomical and visual outcomes of fovea-sparing internal limiting membrane peeling with or without inverted flap technique for myopic foveoschisis. BMC Ophthalmol Nov.

[2] Lin JP, Yang CM (2022). Combined fovea-sparing internal limiting membrane peeling with internal limiting membrane flap technique for progressive myopic traction maculopathy. Graefes Arch Clin Exp Ophthalmol Feb.

[3] Bonińska K, Nawrocki J, Michalewska Z, Mechanism of “Flap, Closure” After the Inverted Internal Limiting Membrane Flap Technique. Retina Nov. 2018;38(11):2184–9. 10.1097/iae.0000000000001861. 10.1097/IAE.0000000000001861 28961670

[4] Tsui MC, Yang CM, Early and Late Macular Changes After the Inverted Internal Limiting Membrane Flap, Technique for a Full-Thickness Macular Hole. Retina Jan. 2021;1(1):20–8. 10.1097/iae.0000000000002796. 10.1097/IAE.0000000000002796 32341284

